# Development and Heredity in the Interwar Period: Hans Spemann and Fritz Baltzer on Organizers and Merogones

**DOI:** 10.1007/s10739-022-09681-w

**Published:** 2022-08-05

**Authors:** Christina Brandt

**Affiliations:** Ernst-Haeckel-Haus, Institute for Zoology and Evolutionary Research, Friedrich Schiller University, Jena, Germany

**Keywords:** History of developmental physiology, History of developmental genetics, Merogony experiments, Nuclear transplantation, Cloning

## Abstract

This article explores the collaborative research of the Nobel laureate Hans Spemann (1869–1941) and the Swiss zoologist Fritz Baltzer (1884–1974) on problems at the intersection of development and heredity and raises more general questions concerning science and politics in Germany in the interwar period. It argues that Spemann and Baltzer’s collaborative work made a significant contribution to the then ongoing debates about the relation between developmental physiology and hereditary studies, although Spemann distanced himself from *Drosophila* genetics because of his anti-reductionist position. The article analyzes how Spemann framed the issues of heredity in terms of an epigenetic principle in the context of his work on the “organizer,” and it explores the experimental dynamics of research on newt merogones carried out by Baltzer in a methodological development of Spemann’s constriction experiments. Finally, these research attempts are discussed as part of a broader “prehistory” of the mid-twentieth century cell nuclear transplantation experiments, which provided the basis for later animal cloning.

## Introduction

Since the classical studies of Harwood (1993) and Sapp (1987), several authors have focused on the specificities in the history of genetics and hereditary studies in Germany in the early twentieth century.^1^ Describing differences in research styles in the genetics communities, Harwood has argued that the newly developing field of “genetics” designated a more “inclusive terrain” in Germany than in the United States (Harwood 1993, p. 6). The spectrum of research approaches went far beyond the narrow problems of transmission genetics or what became called Mendelism; it ranged from such fields as physiological or developmental genetics to approaches of evolutionary genetics that regarded development and evolution as fundamental issues (Harwood 1993, pp. 138–142). However, the exchange of research practices and research concepts between experimental embryology and the newly developing experimental research into heredity has been comparatively little discussed. In this article, I focus on the relationship of experimental embryology and studies of heredity in biological research in the interwar period by exploring the collaborative work of Hans Spemann (1869–1941) and the Swiss zoologist Fritz Baltzer (1884–1974). The still dominant historical picture indicates that both fields became separated in the first decades of the twentieth century.^2^ In his classical book on the history of cytoplasmic inheritance, Sapp, for example, has argued that “embryologists continued to work largely in isolation of genetic research and theories. Many insisted that their aims, concepts, and techniques were fundamentally incompatible with those of genetics.” The “school of Hans Spemann” played, in Sapp’s view, “a leading role in fostering the development of embryology away from genetics” (Sapp 1987, p. 68). It is certainly the case that, from an epistemic point of view, research questions and research practices in experimental embryology and hereditary studies differed considerably at that time. However, as I will show with the example of the work of Spemann and Baltzer, the relationship of practices and concepts in these fields was much more complex than the standard narration of a split between genetics and embryology would imply, all the more so when one has to consider that studies on heredity were far more than *Drosophila* genetics in the German-speaking biological research community at that time.

With this focus, this paper also aims to provide a fresh historical perspective on the work of Hans Spemann. Historical interest in Spemann had increased considerably in the 1990s, when the life sciences were shedding more and more light on the molecular mechanisms of embryonic induction, morphogenesis, and the genetic mechanism of pattern formation in development. Spemann’s notion of a so-called “organizer” (that, around 1920, originated as a description of a specific region in the embryo that was able to induce the neural tube and the axial system) was then described as a “key concept in developmental biology” (Sander and Fäßler 2001, p. 1). Biologists saw Spemann’s experimental work as having initiated a twentieth century research endeavor that, after a few decades of neglect following World War II, reemerged in the 1980s (DeRobertis 1995, 2009). From this view, the results of molecular developmental biology from the 1980s and 1990s were seen against the historical background of shifting paradigms in experimental embryology over the course of the twentieth century (Gilbert and Saxén 1993; Gilbert 2001).

While biologists have tended to discuss Spemann from the perspective of twentieth-century developmental biology, historians of science have analyzed Spemann as a representative figure for the shifting paradigms of experimental embryology from the late nineteenth to the early twentieth century. In history of biology, Spemann is usually described as belonging to those leading scientists who established a material, causal, and experimental approach to embryology at the turn to the twentieth century,^3^ but also as having missed or ignored the new developments in genetics and biochemistry at the time (Oppenheimer 1970; Hamburger 1988; Fäßler 1997; Allen 2007). With his microsurgical approach to the newt embryo and his constriction and transplantation experiments, Spemann (like the US zoologist Ross Harrison and others) devised new experimental techniques and played a leading role in developing new research approaches in embryology around 1900. After Wilhelm Roux’s (1850–1924) pioneering experiments on frog embryos in the 1880s (Roux 1888) and his research program of *Entwicklungsmechanik* [developmental mechanics] that searched for the mechanical causes of differentiation, this new generation of younger scientists pushed forward a new wave of experimental research in embryology, although they often, like Spemann, replaced Roux’s reductionism with a new holism (Hopwood 2009; Mocek 1998, pp. 183–255; Nyhart 1995, pp. 278–305; Maienschein 1991a). Especially in the German context, the attempts in experimental embryology increasingly detached embryological studies from the evolutionary framework developed in the days of Ernst Haeckel. Within this historical context, Spemann’s work on the organizer was part of an attempt to understand organismic regulation. Such approaches often granted a place, as Jane Maienschein has argued, to “vitalistic considerations” (Maienschein 1997, p. 222).^4^

In addition to the lively discussions in history of science as to whether Spemann advocated vitalistic concepts (Allen and Maienschein 1999; Hamburger 1999; Allen 2005), other historians of biology have discussed the question of how neo-Lamarckism in the early 1900s influenced Spemann (Rinard 1988; Fäßler 1997). Nevertheless, most historical studies have focused on Spemann’s experimental work on the “organizer.” These historical studies were interested in the origin of the concept, its role for twentieth century biology (for example, Oppenheimer 1970; Fäßler 1997; Sander and Fäßler 2001, DeRobertis 2009), and its influence on research developments in several countries (DeRobertis and Arechaga 2001). Others have raised questions about issues of priority (Lenhoff 1991) or they have taken a historical perspective that considers the political dimensions of the concept itself (Horder and Weindling 1986).

Despite being widely discussed in previous historical studies from the 1990s and early 2000s, interest in Spemann in the history of biology has diminished in more recent years. This article will continue the historical analysis of Spemann’s work, but it will also broaden the perspective. By focusing on the Spemann–Baltzer collaboration, this article wants to shed new light on Spemann’s relation to genetics and studies of heredity and thereby revise the understanding of the relationship between experimental embryology and hereditary studies in the German-speaking research community of the first decades of the twentieth century. I argue that, although Spemann’s holistic approach (like that of many German zoologists at that time) was in clear opposition to the developing *Drosophila* genetics, this does not mean that he ignored research developments in genetics more generally. On the contrary, Spemann also supported what he called an “epigenetic” view (Spemann 1924a, p. 78) that integrated *Entwicklungsphysiologie* [developmental physiology] and physiological studies of heredity. He thereby aimed to contribute to a research program on development rooted in the work of his former teacher Theodor Boveri (1862–1915). Moreover, by using practical tools of experimental embryology, Fritz Baltzer, Spemann’s first assistant in Freiburg (and later a professor of zoology in Bern) contributed with seminal studies to the newly emerging field of developmental genetics.

In the initial sections of this article, I discuss the 1923 meeting of the German Society for Hereditary Studies [*Deutsche Gesellschaft für Vererbungswissenschaft*] in Munich and raise questions about science and, more generally, about Spemann’s attitude toward politics in the early Weimar Republic. The talk he gave at this meeting (Spemann 1924a, b) is one of the few places in which Spemann developed his theoretical views of heredity and development. In this text, Spemann argued in favor of what he called an “epigenetic principle” (Spemann 1924a, p. 78). In the early 1920s, Spemann not only saw his research on the organizer as a first experimental step toward a deeper investigation of what he then called the “activation of hereditary material” [*Aktivierung der Erbmasse*] (Spemann 1924a, p. 73), but he also referred at length to experiments on the cytoplasm–nucleus relationship carried out in his laboratory by his assistant Fritz Baltzer at that time.

The final three sections of the article explore Spemann and Baltzer’s collaborative research in more detail and analyze their constriction experiments [*Schnürungsexperimente*] and *merogony* experiments with the newt *Triton* (experiments in which an enucleated egg was fertilized by sperm, resulting in a haploid organism, the *merogone*). These experiments are analyzed in the context of the related debates about the role of the cytoplasm and nucleus in heredity, which were widespread in the German-speaking scientific community of the time. This work on the intersection of heredity and development continued a research approach of Boveri. It was within this research tradition that Baltzer’s research group in Bern developed a specific approach to developmental genetics in the 1920s and 1930s. It was also within this research tradition that Spemann envisioned what he then called a “fantastical” experiment, referring to the then technically impossible transplantation of differentiated cell nuclei into enucleated egg cells. Spemann described this in his book *Embryonic Development and Induction*, a work based on the Silliman Lectures he gave at Yale university in 1933 (Spemann 1938, p. 211; see also Spemann 1936, p. 135). This kind of thought experiment by Spemann was later often interpreted as “foreseeing” cloning. Scientists who worked intensively on techniques of cell nuclear transplantation and cloning in the 1960s and 1970s—for example, McKinnell (1979) and Di Berardino (1997)—referred to Spemann’s work as a kind of visionary origin of cloning research (see also Maienschein 2003, pp. 115–118; Crowe 2014, pp. 65–66). In the last part of this article, I will also explore the historical and theoretical context of Spemann’s reference to the “fantastical” experiment. I will show that Spemann did not use this notion primarily as a hypothetical prediction but, more fundamentally, with reference to general research questions about determination and potentialities in the developing organism.

## Munich 1923 – Hans Spemann: Science and Politics in the Weimar Republic

Spemann, then head of the Institute of Zoology at the University of Freiburg, was one of the keynote speakers at the third meeting of the newly founded *Deutsche Gesellschaft für Vererbungswissenschaft* [German Society for Hereditary Studies], which took place in Munich in September 1923. In his talk “Vererbung und Entwicklungsmechanik” [Heredity and Developmental Mechanics], Spemann tackled a central topic of the meeting, namely, the relationship between the rapidly developing experimental studies on heredity and research in developmental physiology. The title of this talk, however, was surprising: Spemann typically avoided speaking of *Entwicklungsmechanik* because of its strong mechanistic connotations. He instead preferred the term *Entwicklungsphysiologie* [developmental physiology]. Already in 1907, Spemann had written to the pioneer in this field, Wilhelm Roux, that he would regard developmental physiology as a “more sympathetic designation” for those studies in experimental embryology.^5^ However, Spemann’s contribution to the Munich meeting was not only exceptional because of the title. The conference was one of the very few, if not the only, public occasions where Spemann, who was well known for both his reluctance to engage in theoretical speculation as well as for his scientific ignorance of the emerging Mendelian genetics, discussed theoretical issues of the new field of hereditary studies at length. Spemann most likely did not prepare his contribution to the meeting at his own initiative but following a request that was hard to refuse by the organizer of the Munich meeting, Richard Hertwig (1850–1937).^6^ Hertwig, who had been a professor at the University of Munich since the 1880s, was the doyen of German zoology, and he was still an active director of the Institute of Zoology in Munich in the early 1920s.

The conference in Munich was overshadowed by the massive economic and political crises of the young Weimar Republic. “The precarious political situation and the increasing economic poverty in summer 1923,” as Richard Hertwig and Hans Nachtsheim (1890–1979) put it in their conference report, rendered it extremely questionable, right up to the last moment, whether the meeting in Munich would take place at all (Hertwig and Nachtsheim 1924, p. 237). Indeed, the year 1923 is described by historians as *the* year of the main crisis, one in which the young democracy was deeply unsettled by internal and external conflicts. Some regions of the country were on the brink of a civil war, and the political conflict with the Allies of World War I, especially with France, escalated after the occupation of the Ruhr. All of this was accompanied by a dramatically escalating economic crisis and massive hyperinflation.^7^

A passing glance at the Munich conference shows the extent to which the political and economic situation also affected scientific research. Founded just two years earlier, the German Society for Hereditary Studies had 350 members in 1923, among them many scientists from other countries. The annual fee for German members rose from 50 *Mark* in January to 50,000 *Mark* (so-called *Papiermark*) in September 1923. It was only because of the international members, as the conference report emphasized, that the financial situation of the society was at all bearable (Hertwig and Nachtsheim 1924, p. 259). The political crisis escalated even further that year when right-wing politicians tried to take political power in Munich and Bavaria, precisely during the week that the scientists met in the city.^8^ The Munich conference is just one small example of how fragile the system was in those years. The political and economic crises in the early Weimar Republic not only deeply affected the external organization and funding of science (as the foundation of the so-called *Notgemeinschaft der Deutschen Wissenschaft* in 1920 reveals, an organization that tried to prevent the financial collapse of the sciences in Germany), but also its internal conditions and the political atmosphere within academia. Many now well-known historical studies have analyzed extensively how, in contrast to the liberal and left-wing views of many intellectuals, artists, and writers outside the university system, conservative and nationalist sentiments were often predominant in the professorate and academic elite of the Weimar Republic.^9^

Hans Spemann also must be regarded as a political conservative, even though he was a strong supporter of reform pedagogical movements and, after World War I, of the *Volkshochschulbewegung* (an adult education program and movement that aimed to educate classes largely excluded from access to learning).^10^ The historian Peter Fäßler (1997) provided a comprehensive biography on Spemann that gives a detailed historical reconstruction of his experimental work from 1900 until the 1930s. He describes Spemann as the sort of professor who avoided making direct political statements and who never belonged to any political party, but who nonetheless had strong patriotic feelings (Fäßler 1997, pp. 86–97). However, the extent to which Spemann’s nationalist attitude became intermingled with his role as a scientist in the political atmosphere of the early years of the Weimar Republic becomes quite clear during the occupation of the Rhineland. In June 1923, as the Rector of the university of Freiburg, Spemann personally led a funeral procession for a militant nationalist activist (Albert Schlageter), who was executed by the French troops in the Ruhr area (Fäßler 1997, p. 89). In January 1925, he explained in a letter to the British polymath Julian Huxley (1887–1975) that he had refused an invitation from the British geneticist William Bateson (1861–1926) for political reasons:

Dear colleague! I just have written to Bateson that I will be unable to come this spring. Too much work and then – You also would not give a lecture in Germany if we were to continue to occupy a part of England and refused to leave at the appointed time. Visit me here one day. With kind regards, yours faithfully H. Spemann.^11^

At the time of the conference in Munich, the academic career of the 54-year-old zoologist was already at its peak. In 1919, Spemann had succeeded August Weismann (1834–1914) as the holder of the prestigious chair for zoology in Freiburg, and already in the 1923/1924 academic year he had been elected Rector of the university.^12^ When he accepted the offer in Freiburg, Spemann resigned from the directorship of a department at the Kaiser Wilhelm Institute for Biology in Berlin, where he had spent the years during World War I. He had been a part of the first generation of directors, together with Carl Correns (1864–1933) and Richard Goldschmidt (1878–1958), of the relatively new institute, which had been founded in 1914.

After his Berlin years, when Spemann’s research seemed to be developing slowly because of the difficult wartime situation and the work surrounding the establishment of a new institute, the situation changed considerably in Freiburg in the early 1920s. His scientific work began to flourish and attracted a growing group of research students. Within a few years, he had established his laboratory as an attractive venue for scholars from abroad. Spemann’s most successful research period—his work on the so-called “organizer”—was during these Freiburg years. In June 1923, three months before the Munich conference, Spemann had submitted to *Roux’ Archiv für mikroskopische Anatomie und Entwicklungsmechanik* a co-authored article on the pioneering findings of his PhD student Hilde Mangold (née Pröscholdt, 1898–1924) on the famous organizer experiments. The article, “Über Induktion von Embryonalanlagen durch Implantation artfremder Organisatoren” [On the induction of embryonic primordia by implantation of organizers from different species] was published a year later in September 1924 (Spemann and Mangold 1924). Already, from 1915 to 1917 at the Kaiser Wilhelm Institute in Berlin, Spemann had carried out homo- and heteroplastic transplantation experiments on the differently pigmented newt embryos *Triton taeniatus* and *Triton cristatus*, work that he continued at Freiburg. In Berlin, he had observed that tissue from a specific region in the gastrula state of the embryo, the dorsal blastopore lip,^13^ produced a secondary axial structure when transplanted into the ventral region of a host embryo. Initially, he had interpreted this as showing that these embryonic structures derived from the transplanted tissue. 14 In the years 1921 and 1922, Hilde Mangold carried out more than two hundred such transplantation experiments. A few but significant number of them came to the surprising result that the secondary axial structures consisted of chimeric tissue from the graft *as well as from the host*, whereby most of the tissue of the newly formed embryonic structures was derived from the host embryos (for detail, see Fäßler 1997, pp. 249–264). Spemann and Mangold therefore concluded that the dorsal blastopore lip was able to form an entire secondary embryo by arranging the host tissue into a secondary axial system. Because of this, they called this specific region of the developing embryo an “organizer” and assumed that it had the fundamental potential to direct the embryo’s development (Spemann and Mangold 1924).

Spemann’s former colleague at the Kaiser Wilhelm Institute, Richard Goldschmidt, later reminded Spemann that he had called the microsurgical transplantation a scientific “gold mine” already during his time at the Berlin institute.^15^ Spemann was in fact able to turn his approach into a highly dynamic experimental system during the years that followed in Freiburg (Spemann 1924c, 1927a, b, c, 1929; Spemann and Schotte 1932). In the 1920s and early 1930s, not only did his growing research group investigate different spatial and temporal aspects of organizer effects in a highly systematic way, but a kind of “gold rush” was underway in many international laboratories in research on the organizer. Spemann was often regarded as “the leading European zoologist” among his contemporaries.^16^ He was awarded the 1935 Nobel Prize in Physiology or Medicine for his research.

Despite Spemann’s new and highly dynamic research focus, and his group’s specialization in the investigation of the “organizer” in Freiburg in the 1920s, he also continued his “older” research on constriction experiments on newt embryos—experiments that were rooted in Spemann’s Würzburg period when he worked with Boveri. It was this research trajectory that provided the background to Spemann’s talk in Munich in September 1923. What does this talk tell us about Spemann’s theoretical views at that time?

## Spemann’s Epigenetic Approach: “Activation of Heredity Material” and Antireductionism

It is often claimed in the historical literature that Spemann, who almost never used the term “gene,” ignored new trends in early twentieth century genetics, especially the results from Thomas Hunt Morgan (1866–1945) and his group. For example, Fäßler (1997, pp. 310–314) raised the question of whether Spemann had contributed to a split between genetics and developmental biology in the early twentieth century. According to Fäßler, Spemann’s lack of theoretical interest in the new trends in genetics resulted from the fact that his own discipline—developmental physiology—was a highly dynamic and specialized field at that time and that Spemann’s fundamentally empirical research approach prevented him from speculating about theoretical but as yet unproven assumptions.

Spemann’s contribution to the Munich conference is very illuminating with respect to this issue. Spemann’s 1923 talk is a rare document in that he explicitly and at length discussed theoretical issues concerning the intersection of heredity and development. It was published a year later (as part of the conference report), not just in the German journal for new studies in genetics, the *Zeitschrift für inductive Abstammungs- und Vererbungslehre* (Spemann 1924b), but also in *Die Naturwissenschaften* (Spemann 1924a), a weekly general science journal that claimed to be the German equivalent to *Nature.* The article was thus widely received as documenting the state of research in contemporary biology for a broad scientific audience.

By theorizing about the relationship between physiological genetics and developmental physiology, Spemann’s article clearly indicates that some of the lines of his own experimental work did indeed resonate with theoretical developments in hereditary studies in a broad sense. This held both of what were then perceived as being promising experimental tools and studies for investigating issues of heredity as well as of the question of what *heredity* meant in the German research of that period. Moreover, the article shows that the scientific validity of the very notion of “inheritance” itself was still being discussed by scientists as late as 1923. Spemann pointed to the semantic limitations of the term *Vererbung* [inheritance].^17^ This was not only meant as a rhetorical move; it was also central to the holistic approach characteristic not just of Spemann but also of many of his colleagues who saw issues of heredity as only a smaller aspect of the then contemporary all-encompassing organismic perspective. At the level of the whole organism, the narrow concept of heredity would lead into the broader problem of development. However, by arguing in this way, Spemann did not seek to reject experimental studies on heredity. On the contrary, he not only addressed what was seen as the main research problems at the intersection of genetics and developmental studies, but also situated his own research activities within this research domain. He discussed at length questions concerning the localization of heredity factors, the problem of their nature, and the mode of action of genes, which Spemann framed as the problem of the “activation of the hereditary material” [*Aktivierung der Erbmasse*] (Spemann 1924a, p. 73). There are three important aspects to this text, published in 1924, that should be emphasized.

First, the historical discussion of Spemann’s lack of interest in genetics tends to imply that he did not belong to the “modernists” but had an approach to science characteristic of “older” embryological research. However, the situation is the opposite: In the eyes of many of his contemporaries, at least in the German-speaking research community, Spemann was seen as representative of a modern way of doing biology precisely because he distinguished his research from physicochemical reductionism.^18^ It is true that Spemann largely ignored the results of the *Drosophila* research of the Morgan school, but for reasons different from those noted above. Spemann interpreted Morgan’s results not primarily as a new and promising approach in biology but, on the contrary, as the continuation of a *previous*, almost rejected reductionist research tradition. Specifically, Spemann described Morgan’s work as a continuation of Weismann’s notion of determinants [*Determinanten*] localized in the germplasm. This is stated clearly in his comparison of Morgan’s gene maps with a representation in the style of Weismann: “a factorial topographical map of chromosomes from Morgan looks like Weismann’s schema but filled in with concrete content.”^19^ Thus, Spemann did not see Morgan as a representative of the future of genetics but as belonging more to a previous reductionist research paradigm. A different tradition of studies on heredity provided for Spemann the main research models in the field, particularly those in the tradition stemming back to his own teacher and mentor Theodor Boveri. However, he also extensively discussed the work of his former colleague at the Kaiser Wilhelm Institute for Biology, Richard Goldschmidt, as an outstanding example of a productive alliance between Mendelian research and developmental physiology. Goldschmidt developed an approach in which genes were regarded as enzymes or enzyme-like entities. His research interests were not concerned with their corpuscular character or with the transmission of genes, but rather with the processes in which the interaction of these entities with other reactions produced effects on physiological activities.^20^ This physiological approach, which Spemann called the “enzyme-hormone-theory” (1924a, p. 67), focused on the processes at the intersection of heredity and development and described hereditary elements as being only parts of a manifold network of physiological and biochemical reactions. It fit very well with Spemann’s organism-centered view.

Second, Spemann strongly emphasized the notion of “activation of hereditary material” (1924a, pp. 73, 78) and stressed an “epigenetic principle” (1924a, p. 78), which he distinguished from a narrow reductionist perspective. Central to the theoretical framing of this issue was his differentiating an older evolutionary paradigm going back to Weismann from what he described as an “epigenetic” new paradigm. For Spemann, Weismann’s old notion of hereditary factors as the sole and independent “determinants” of hereditary processes and the exclusive controllers of cell differentiation in development had been disproven, and with this the understanding of these processes regarded as an “extremely delicate but rigid clockwork mechanism.”^21^ For Spemann, these views had been scientifically rejected, partly by his own constriction experiments from around 1900 that had challenged the so-called mosaic theory, according to which the process of cell-differentiation in development was explained by a gradual transformation of the nuclear substance and in terms of a mechanistic process of parceling out determinants to different cells.^22^ In contrast to Weismann’s idea of the action of determinants or—in the vocabulary of the early 1920s, *genes*—Spemann emphasized the “activation of the hereditary material” as a “complex network of interactions,” and an interplay in different “steps of development” (1924a, p. 67).

Spemann’s choice of words is very interesting: When he described Weismann as an “extreme evolutionist,”^23^ he used the word *evolution* with the longstanding meaning from the period around about 1800, when the German word *Evolution* was practically synonymous with *preformation*. The term *epigenetic*, which Spemann understood as self-evidently referring to a modern, non-reductionist view of heredity and development and which also had its roots in the debates around 1800, had certainly acquired a new meaning for his contemporaries when the new field of genetics became established and after the introduction of the term *gene* to the biological vocabulary more than a decade earlier.

Third, Spemann’s text clearly shows that in the early 1920s, he certainly saw his own research on the “organizer” as contributing to these broader problems at the intersection of heredity and development, particularly to the investigation of the “activation of hereditary material.” Furthermore, he referred at length to research carried out in his Freiburg laboratory—following the tradition of his former teacher Boveri—by his assistant Fritz Baltzer on the problem of the interaction between the cytoplasm and the nucleus in hereditary processes. In later writings, Spemann never again broached theoretical issues of heredity in such detail. However, Spemann’s colleagues also realized how closely connected his research was to problems in experimental work in the broad field of genetics. In early 1923, Spemann and his former colleague at the Kaiser Wilhelm Institute, the botanist and geneticist Carl Correns, discussed possible interpretations of Spemann’s new results concerning the “organizer” effect. Correns, well-known as one of the “re-discoverers” of Mendel in 1900, favored a cytoplasmic view of heredity and saw the cytoplasm as the constitutive part and regulatory structure of gene activities. Correns sometimes used an unorthodox metaphor in his lectures and publications by comparing the gene–cytoplasm relationship with a machine gun, a metaphor that in the wake of World War I drew on a familiar experience. Correns metaphorically described the genes as the “bullets” of a machine gun, where the “gun” (seen as the more complex and active part of the system that ordered the gene activities) was, for Correns, located in the cytoplasm.^24^ Correns soon connected his machine gun analogy with Spemann’s new concept of the organizer as a superordinate structure. In February 1923, he wrote to Spemann:

I am VERY [sic] interested in what you write about the “Organizers.” If I understood you correctly, the bigger taeniatus piece receives the (continuous?) impulse for development from the smaller cristatus-piece, but remains species-specific, like the latter. This is the contrast between that which one assumes are genes localized in the chromosomes and that, what arranges the unfolding [*Entfaltung*] of the genes in the right order, the bullets and the machine gun. Do I understand you correctly?^25^

Thus, Spemann took note of what his former colleagues from the Kaiser Wilhelm Institute for Biology researched in the rapidly developing field of genetics, and they were also aware of his research in developmental physiology. Correns and Spemann exchanged letters long after Spemann had left the Berlin institute. As Correns’s keen interest in Spemann’s concept of the “organizer” shows, in the early 1920s Spemann’s work was regarded as being highly compatible with the then dominant genetic discourses on cytoplasmic inheritance in Germany.

## Boveri’s Legacy and the Collaborative Research of Spemann and Baltzer

In the early 1920s, Spemann was interested in understanding phenomena at the intersection of heredity and development even though he was, like many of his German colleagues, highly skeptical of the newly developing *Drosophila* genetics and its underlying epistemic assumptions.^26^ Spemann continued to regard his former teacher Boveri as a leading figure in hereditary studies. Boveri’s experiments on chromosomes, especially his merogony experiments, provided Spemann in the early 1920s with a guiding experimental paradigm for tackling problems concerning the activation of hereditary factors. The research dynamics of Baltzer’s work in Spemann’s laboratory in Freiburg around 1920 can only be understood against the background of this research tradition.

Boveri’s research on sea urchins concerning questions of fertilization, chromosome individuality, and the role of the cell nucleus in development had made him one of the most prominent biologists in Germany in the first decade of the twentieth century. Baltzer, then a 21-year-old student, came to study with Boveri in Würzburg in 1905 (Lehmann 1954, p. 1; Rosin 1975). Just a year before, in 1904, the 35-year-old Spemann had become an extraordinary professor [*außerordentlicher Professor*] at the Institute for Zoology in Würzburg with Boveri’s support. Spemann had already completed his PhD under Boveri’s supervision in 1894 and finished his *Habilitation* in 1898. It was during these years in Würzburg that Spemann had performed his first, now well-known, constriction experiment [*Schnürungsversuche*] on newt eggs, whose findings challenged Roux and Weismann’s so-called “mosaic theory” (Spemann 1901, 1902, 1903).

In the early years of the twentieth century, Spemann was already regarded as a promising scholar in the German zoological community. This led to his first professorship. In 1908, he moved to the University of Rostock as a full professor, where he stayed until 1914, the year he became the second director at the newly founded Kaiser Wilhelm Institute for Biology in Berlin.^27^ It was again Boveri who pulled the strings for this appointment. When the Prussian state started to plan a Kaiser Wilhelm Institute for Biology, Boveri was considered for the position of the director and had a major influence in shaping the research orientation as well as personnel decisions. Boveri ultimately did not accept the directorship because of health concerns. As a result of complex negotiations, the botanist Carl Correns (hereditary studies and genetics) was appointed as the first director of the newly founded institute; Spemann was appointed the second director and the head of a department for *Entwicklungsmechanik*; Richard Goldschmidt (animal genetics), Max Hartmann (protozoology), and Otto Warbug (physiology) became heads of departments (Sucker 2002, pp. 151–198).

During Spemann’s years in Rostock and Berlin, Baltzer stayed with Boveri in Würzburg. He researched sea urchins and the marine worm *Bonellia*. His PhD thesis was on questions about mitosis (Baltzer 1908a). As Boveri’s assistant, he mainly worked on chromosomes and inheritance and on problems concerning sex determination (Baltzer 1908b, 1910, 1911, 1914a). He explored whether the chromosomes of one sea urchin species could stimulate development when transferred to the cytoplasm of another species. Even though Baltzer was deeply concerned with questions about inheritance, and even though he focused on the role of the nucleus, he was nonetheless very careful to avoid taking a fixed position on the emerging debate between the neo-Lamarckian and the new Mendelian geneticists, as can be seen in his 1914 review of the research on the inheritance of acquired characteristics (Baltzer 1914b). When Boveri died suddenly in 1915, Baltzer’s decade of fruitful research in Würzburg came to an abrupt end, although he remained as an extraordinary professor [*außerordentlicher Professor*] at the university (Lehmann 1954, p. 2). When Spemann took over the chair of zoology in Freiburg in 1919, he offered Baltzer a position. For two years, until 1921, Baltzer worked as Spemann’s first assistant.^28^ These years laid the basis for an experimental approach that in the following years became a productive experimental system. The biologist Viktor Hamburger (who himself belonged to the first generation of PhD students in Freiburg) later gave a very lively picture of the working atmosphere at that time, describing not only Baltzer’s classes in genetics and cytology but also the group’s leisure activities, such as skiing in the Black Forest (Hamburger 1984, p. 10).

In 1921, Baltzer left Freiburg and became professor and the director of the Institute of Zoology at the University of Bern. However, Spemann and Baltzer continued to collaborate in these years. The depth of their shared personal commitment to Boveri’s legacy becomes clear from their writings. In February 1916, shortly after Boveri’s death, Spemann, describing himself proudly as Boveri’s “first and oldest student” (Spemann 1916, p. 11), wrote a personal obituary for the *Physikalisch-Medizinische Gesellschaft* in Würzburg.^29^ Even decades later, the Nobel Laureate Spemann dedicated his main work, the book *Experimentelle Beiträge zu einer Theorie der Entwicklung*, to Boveri (Spemann 1936).^30^ After he had retired, Baltzer wrote a book-length biography of Boveri, praising his personality and the scientific legacy he bestowed to the twentieth century (Baltzer 1962).

These shared feelings of belonging to the same (past) research tradition seem to have provided the basis for a personal commitment to the future. As their letters clearly show, Spemann and Baltzer developed a long-lasting scientific friendship.^31^ In the 1920s, the Institute of Zoology in Bern was clearly an important point of connection in the developing scientific networks around Spemann. In addition to mutual research interests, exchanges of letters, and mutual visits, Spemann and Baltzer also exchanged students. In 1941, Baltzer explained that the Institute of Zoology in Bern has a deep “debt of gratitude to Spemann” because almost all of the researchers “had been his students, either directly or indirectly in the second generation.”^32^ Fritz Lehmann, who completed his PhD in Freiburg in 1926 and subsequently worked as an assistant in Bern, is a good example of this. Gerhard Fankhauser, a Swiss zoologist who worked with Baltzer in the 1920s on Spemann’s constriction method and later became professor of zoology at Princeton (Fankhauser 1972), draws a similar picture of the overlap between Baltzer and Spemann’s research.

What kind of experiments did Baltzer and Spemann develop in Freiburg? On the practical side, they continued following the logic behind Boveri’s famous so-called merogony experiments. This gained a new relevance in the context of the theoretical developments after World War I. In Freiburg, Baltzer combined the rationale of Boveri’s approach with Spemann’s well-established model object (*Triton*) and his sophisticated research methods concerning the constriction of *Triton* eggs. Before turning to these 1920s experiments in more detail, a closer historical look at Boveri’s earlier hybrid merogone experiments and Spemann’s constriction experiments is needed.

The famous merogony experiments, which Boveri had performed in 1889 and 1895, are usually regarded as an early experimental attempt to prove the dominance of the nucleus in heredity. Boveri, whose research laid the basis for the chromosome theory of heredity, worked with sea urchin eggs. Through shaking unfertilized eggs, he produced enucleated fragments of eggs that were then fertilized with sperm of different sea urchin species. The resulting haploid organisms—the so-called merogones—apparently only had characteristics from the paternal lineage. These experiments were soon regarded by Boveri and his contemporaries as proof for a nuclear dominance in development—despite the objections of colleagues such as T. H. Morgan, Hans Driesch, and Yves Delage in the 1890s (Laubichler and Davidson 2008, p. 5; see also Sander 1997). However, Boveri himself became skeptical of his results toward the end of his life. When he and his wife Marcella O’Grady repeated similar experiments in Naples in 1911/1912 and 1914, it became obvious that they could not exclude the possibility that the egg fragments still contained parts of maternal chromosomes (Boveri 1918). Helga Satzinger has discussed in detail how these later results greatly unsettled Boveri’s former belief in the dominance of chromosomes in heredity (Satzinger 2009, pp. 113–123; see also Satzinger 2008). Boveri also attempted to create better research techniques. Satzinger refers to a letter to Spemann of November 1913, in which Boveri wrote that he planned to develop an experiment for extracting single chromosomes from the sea urchin egg by using tiny capillary tubes, which would allow him to investigate the developmental role of these chromosomes.^33^ However, in contrast to the enthusiasm generated by the new focus on genes, Boveri, like many German zoologists, remained skeptical of the explanatory power of Mendelian genetics. In his view, this approach could only investigate superficial gene effects. Despite the noticeable scientific success in that new field, he saw its limitation. In an article published posthumously in 1918, Boveri wrote: “About heredity itself, however, that is, the question of how the given constellation within the zygote leads to hereditary effects … we know almost nothing” (Boveri 1918, p. 465).^34^

**Fig. 1:**
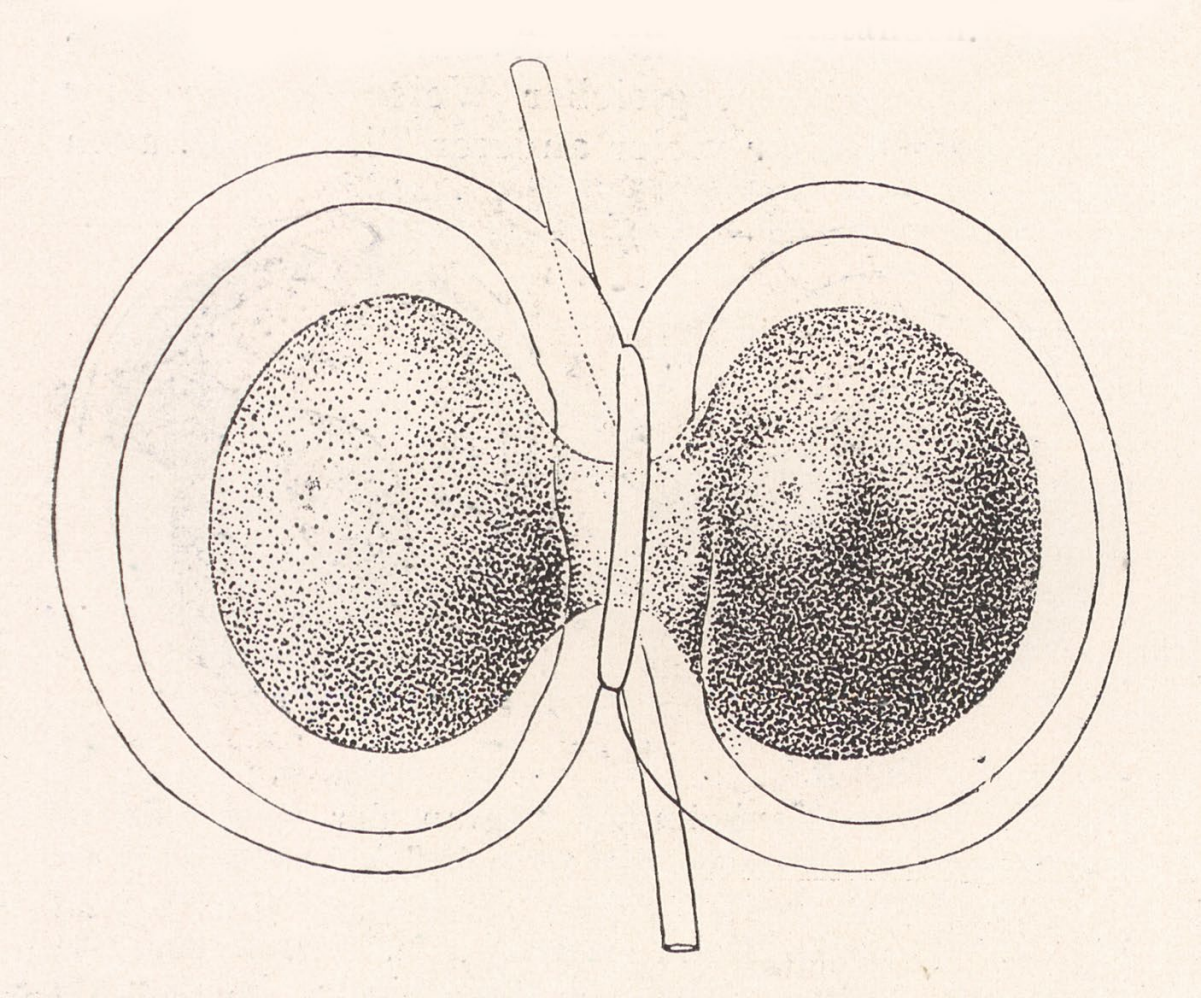
The basic method of Hans Spemann’s constriction experiments, using a “hair loop”. The picture shows a constricted egg of the newt (*Triton taeniatus*) shortly after fertilization. (From Spemann 1919, p. 582, reprinted by permission from Springer Nature, The Science of Nature)

Since Spemann and Boveri were exchanging letters at that time, Spemann probably knew in or around 1913 that Boveri was questioning his own earlier results in the merogony experiments. This was also the period in which Spemann began to modify his earlier constriction experiments from the late 1890s. Whereas Spemann’s early experiments in Würzburg were performed in the context of the debate between Hans Driesch (1867–1941) and Wilhelm Roux about the embryo’s capacity for self-regulation, in 1913 Spemann modified his experiments in Rostock. The focus shifted toward questions concerning the nucleus–cytoplasm relationship. In his earlier experiments from the late 1890s, he had, by using a hair loop, constricted the early embryo of *Triton taeniatus* in the 2- or 4-cell stage as well as at the gastrula and even later developmental phases Spemann (1901, 1902, 1903; see also Fäßler 1997, pp.152–186). Constriction of the blastomere in the early stages resulted in the normal development of twin embryos or of double-headed creatures; constrictions in later phases failed to produce embryos that developed normally. In summer 1913, however, Spemann began to constrict newt eggs immediately after fertilization and *before* they started cleavage (Spemann 1914, p. 216). By using the hair loop method, he divided the fertilized egg into two halves that remained connected through a thin plasma bridge (see Figure 1). Whereas the nonnucleated half remained in its original condition, the part that contained the (fertilized) egg nucleus underwent cleavages. After a number of cleavages, one of the resulting nuclei migrated through the thin plasma bridge into the nonnucleated half—a process that happened sooner or later—and this part also underwent cleavage. As a result, Spemann produced twin embryos at different temporal stages. He observed that even the nuclei that resulted from the sixteenth cell division [*1/16. Furchungskerne*] had, when migrating to the originally nonnucleated part, the potential to stimulate the anucleated half to develop a normal embryo. These results clearly provided a basis for rejecting the mosaic theory and Weismann’s hypothesis of unequal nuclear divisions and irreversible differentiation of the capacity of the cell nuclei during development.

In the 1880s, Weismann had discussed a mechanical model for heredity and development. Weismann speculated that in the process of cell differentiation, there is a successive parceling out of the structure of the germ plasm. He elucidated this process by using a striking analogy: The “development of the nucleoplasm during ontogeny” was compared by him to “an army composed of corps, which are made up of divisions, and these of brigades, and so on.” Taking the “whole army … to represent the nucleoplasm of the germ-cell,” Weismann described the processes of cell differentiation in the following way:

the earliest cell-division as into the first cells of the ectoderm and endoderm may be represented by the separation of the two corps, similarly formed but with different duties: and the following cell-divisions by the successive detachment of divisions, brigades, regiments, battalions, companies, etc.; and as the groups become simpler so does their sphere of action become limited. (Weismann 1892, p. 230–231; English translation in Churchill 2015, p. 306)

If Weismann’s notion of an unequal distribution of nuclear elements at each cell division [*erbungleiche Teilungen*] had been correct, Spemann’s experiments should only have resulted in abnormal embryos—but this did not happen.

However, another observation was even more important for Spemann’s interpretation of the results of his 1913/1914 constriction experiments. This confirmed an assumption that Boveri (1918, pp. 465–466) also had emphasized, namely, that something in the cytoplasm of the egg had a determining influence on early development. Spemann observed that the potential of the constricted halves for development into normal embryos was generally dependent on the *spatial* conditions of the constriction itself. Only after median constriction could the dorsal as well as ventral half develop into normal twin embryos. However, after frontal constrictions, the ventral part was not able to develop normally even if it was the part with the nuclei. For Spemann, this was decisive proof of the importance of the egg cytoplasm. Thus, it is important to stress that for Spemann, the results of his experiments did not primarily concern the remaining capacity of the nucleus in the early phases of development (which, of course, was also shown by his experiments and which contradicted Weismann’s model of unequal nuclear divisions). Rather, this result was of subordinate importance in Spemann’s own theoretical framework, as his focus was not on the nuclei as main actors in development but on the regulatory role of the cytoplasm. Spemann concluded in 1914: “The parts of the embryo developed by the parts of the fertilized egg depend on the egg plasm, not nuclei.”^35^

## Merogones, Transplants, and Hereditary Nuclei in the 1920s and 1930s

Throughout his scientific career, Spemann retained this view of the dominance of the cytoplasm. He had distanced himself from Weismann’s approach and regarded a narrow focus on the cell nucleus or heredity material alone as scientifically unsatisfactory. His interest was in the “activation of hereditary material,” which could only be understood as an “epigenetic principle” and an interplay of the different parts within a complex system (Spemann 1924a, p. 78).

Baltzer, who had worked extensively on chromosomes during his time in Würzburg, seems to have shared this holistic view. However, his experimental approach complemented Spemann’s in that his experiments enabled a focus on the behavior of the nucleus within this system. When Baltzer started to work with Spemann in Freiburg, he changed his research object, turning from sea urchins to the newt *Triton.* Baltzer productively combined the logic behind Boveri’s merogony experiments with the experimental skills developed by Spemann. “Following a suggestion by Spemann,” as Baltzer himself emphasized in 1920, he started to use the method of constrictions on the *Triton* eggs *before* the cleavages in order to produce enucleated egg cells (Baltzer 1920, p. 217). There were two characteristics of *Triton* as a research object that enabled Baltzer to apply the rationale behind Boveri’s merogony experiments to the newt: its polyspermic fertilization and the fact that the fusion of a sperm nucleus with the egg nucleus was a very slow process. Thus, after fertilization each *Triton* egg contained a number of observable sperm nuclei before one of these fused with the egg nucleus. By applying Spemann’s constriction method, it was possible to produce egg halves that no longer contained the egg nucleus but retained one or more sperm nuclei. Baltzer used this to produce *hybrid Triton* merogones. He fertilized the eggs of one newt species (*T. taeniatus*) with the sperm of different species (*T. cristatus*, *T. alpestris*, and *T. palmatus*) (Baltzer 1920). After constriction, these hybrid and haploid embryos developed up to the larval stage and, in one case, Baltzer was even able to produce a *Triton* merogone that developed up to metamorphosis.

The experiments Baltzer carried out in Spemann’s Freiburg laboratory should be discussed within the context of the debates at that time. The question then was no longer whether hereditary material was located in the nucleus. Rather, the debate focused on questions of the relevance of this scientific fact for understanding the processes of heredity and their interplay with development. The active role of the cytoplasm was a central issue, and not just for zoologists who worked in the field of embryology. The lecture that the botanist Hans Winkler (1914) gave at the Munich meeting of the German Society for Hereditary Studies shows that the “interest in cytoplasmic inheritance intensified after the First World War” (Harwood 1993, p. 65; Winkler 1924). Winkler introduced the term *genome* (for the system of chromosomes and cytoplasm) in the early 1920s (Cristescu 2019). The question of the kind of contribution the cytoplasm made to the processes of heredity became a matter of central contention. There was a wide spectrum of different positions on this matter, ranging from the assumption that there were “plasma genes” or the so-called “plasmon theory” (such as that of Correns), which regarded the cytoplasm as a trait-constituting structure, or other views concerning the active, regulatory function of the cytoplasm.^36^

Merogony experiments also underwent a renaissance in the 1920s because of Boveri’s own revisions of his earlier experiments, posthumously published in 1918. A variety of new merogony approaches were developed that had different research objects and different methods for enucleating egg cells. The geneticists Victor Jollos and Tibor Peterfi (from the Kaiser Wilhelm Institute for Biology in Berlin) attempted to produce axolotl merogones by microsurgically removing the egg nucleus (Jollos and Peterfi 1923). Paula Hertwig (the daughter of Oscar Hertwig and the niece of Richard Hertwig) used radium to enucleate frog egg cells and the newt *Triton*. After fertilizing these with sperm from different species, she obtained *Triton* merogones that survived for a period of over 18 days (Hertwig 1923; Landauer 1924). The production of enucleated egg cells was not the only challenge. The (haploid) merogones produced did not survive beyond the larval stage. When Baltzer obtained a *Triton* merogone that developed up to metamorphosis, this was regarded as a spectacular achievement. However, this merogone also died in the end. In October 1922, Baltzer reported news from his institute in Bern to Spemann:

I have to report on the merogone: It was so kind to begin to metamorphose & presented itself quite lively at the Swiss science conference here. However, it then protested and drowned. It was probably too foolish to leave the water after his gills had shrunk, & we were too foolish to take away the water early enough.^37^

On the basis of the findings from his hybridization experiments with merogones, Baltzer concluded that the cell nucleus not only contains species-specific hereditary factors, but also factors that were basically genus specific. Because the cytoplasm always remained the same, yet sperm nuclei from different species induced cleavages, and because of the fact that the development of hybrid merogones always terminated at a specific developmental stage, Baltzer’s interpretation was that the hereditary material in the nucleus remained the same to a certain (and species-variable) extent for *all Triton* species (Baltzer 1920, p. 219). The so-called “*Erbstockhypothese*,” developed by the Jena zoologist Ludwig Plate in the 1920s and early 1930s (Plate 1932; Levit and Hoßfeld 2006), was echoed in Baltzer’s notion of a “graduated equivalence of the nuclear material” [*abgestufte Äquivalenz des Kernmaterials*] (Baltzer 1920, p. 219; 1930, p. xi). Plate differentiated between the “*Mendelstock*,” the Mendelian genes that control species characters, and the “*Erbstock*,” a complex genetic structure that controls the organization and *Baupläne* [blueprints] of genera and higher taxa. Baltzer concluded from his experiments that in the first phase of ontogenesis, those heredity factors that a species shared with other species of the same genus were activated, whereas species-specific heredity factors came into play in later phases. Precisely because of these developmental and phylogenetic aspects, Baltzer’s experimental system had advantages over *Drosophila* genetics. Whereas the latter could only investigate differences between genes and was only concerned with superficial traits (which, for most zoologists, just represented tiny characteristics that had no real value in a taxonomic sense), Baltzer emphasized that his approach aimed to explore the whole genome and that it provided a tool for the experimental analysis of how the basic processes of organ development were guided by heredity material (Baltzer 1940, p. 199).

During the 1920s and 1930s, a very dynamic experimental system on *Triton* merogones was developed in Bern that contributed to the emerging approaches of developmental genetics, although Baltzer himself apparently did not make conspicuous use of this term. In particular, Baltzer’s students Gerhard Fankhauser (who later went to Princeton) and Ernst Hadorn (who completed his PhD in 1931 and his *Habilitation* in 1935 under Baltzer’s supervision) advanced this research. Already in 1926, Baltzer and his group, following the interest in the analysis of organ development, had started combining the work on *Triton* merogones with transplantation experiments, again using another of Spemann’s methods (Baltzer 1940, p. 180). The merogones that always stopped at a specific stage in development were stained, and the scientists transplanted tissue parts of these merogones into normal developing newt embryos. Using this procedure, they tracked how the embryonic tissue (which had originated from a [haploid] sperm nucleus) behaved when embryonic development continued normally (through transplantation into a normal developing embryonic environment) (Baltzer 1930).

**Fig. 2:**
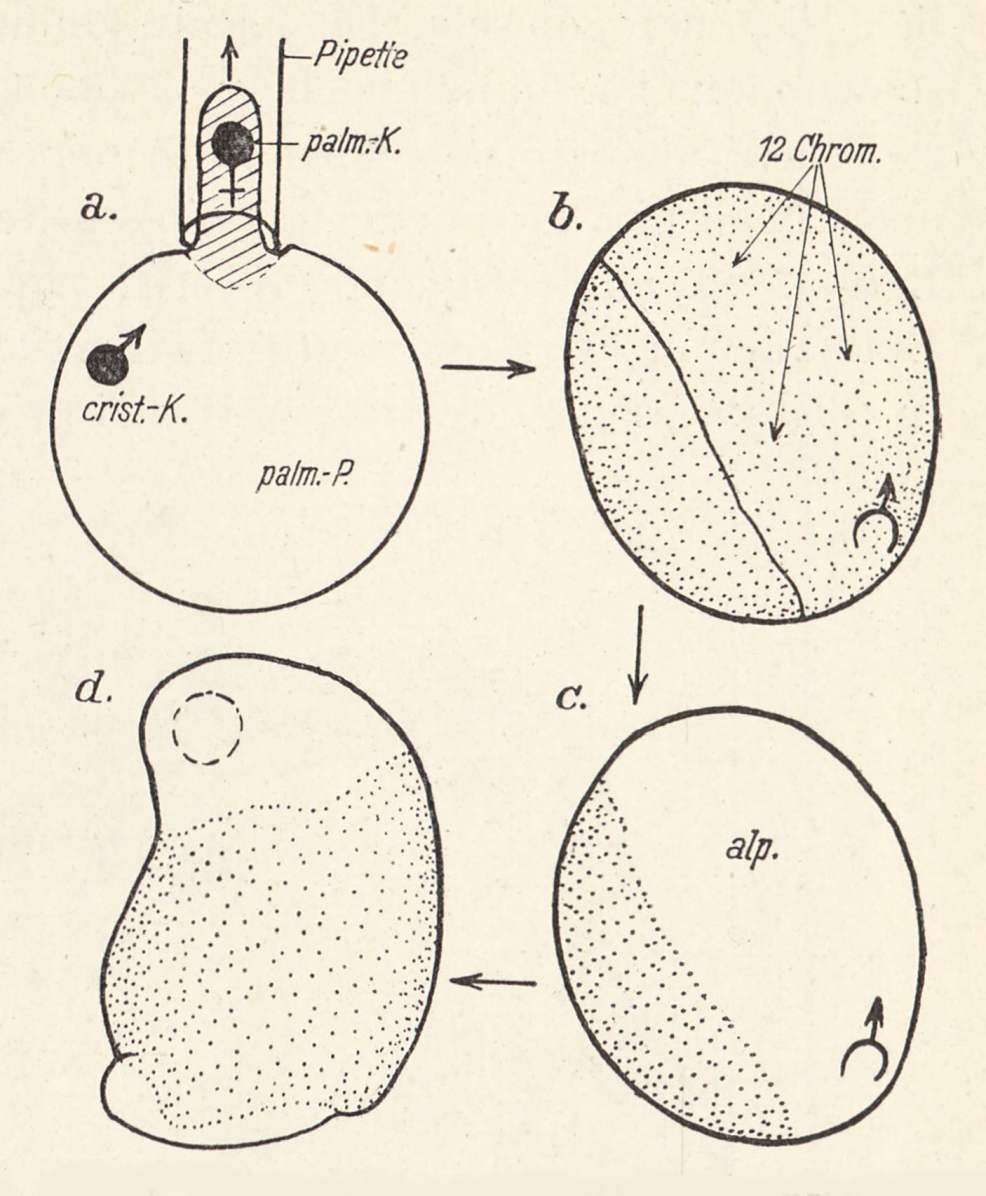
Ernst Hadorn’s hybrid merogone experiments, producing a clear maternal effect: a) Fertilization of the newt egg (*Triton palmatus)* with sperm of a different species (*Triton cristatus*) and removal of the egg nucleus (*palmatus*). The resulting merogone consists of *palmatus* cytoplasm x *cristatus* nucleus. b + c) Tissue from the gastrula of the developing merogone was transplanted into another newt embryo (*Triton alpestris*). d) Proliferation of the hybrid merogone ectoderm (pigmented). (From Hadorn 1936, p. 99.)

**Fig. 3:**
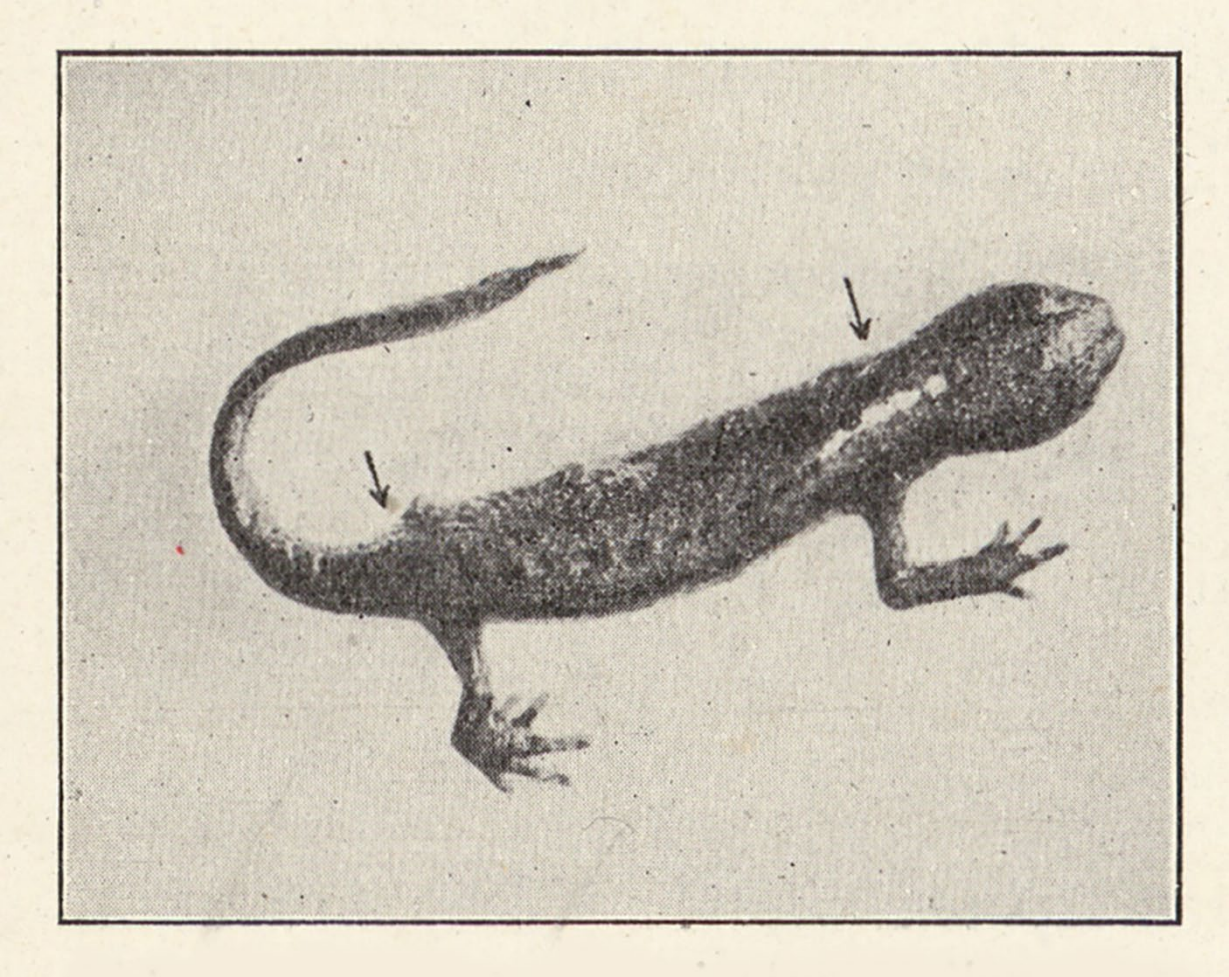
After metamorphosis: *Triton alpestris* with parts of a hybrid merogone epidermis that showed the characteristics of the (female) species type (*palmatus*). (From Hadorn 1936, p. 99.)

Ernst Hadorn, in particular, developed this merogony experiments into a complex system of different hybridization and transplantation experiments. While working in Bern, Hadorn produced a large number of *Triton* merogones from the hybridization of different *Triton* species and used these merogones for several series of tissue transplantations. One surprising result emerged from these experimental series that challenged a nucleus-centered view of heredity: it showed a clear maternal effect. After transplantation of merogone tissue into a host newt embryo, the resulting newt developed (after its metamorphosis) an epidermis that clearly differed from the (male) species type and that instead had the characteristics of the (female) species type (see Figure 2 and 3) (Hadorn 1934, 1936; Baltzer 1940). However, Hadorn changed his research model from amphibia to *Drosophila* after his 1937 visit, supported by Rockefeller funding, to George Beadle’s group in the United States. In the early 1940s, he became director of the Institute of Zoology in Zurich (Nöthiger 1977).

In the 1920s, after Baltzer had left the group, Spemann continued experiments on the fertilization and constriction of *Triton* eggs in Freiburg, even though most of his students were preoccupied with intense research on the “organizer.” In contrast to Baltzer, Spemann remained focused on the cytoplasm. In 1922, Spemann wrote to Baltzer that he had assigned a PhD student to carry out constriction experiments on *Triton* eggs to explore the processes particular to the enucleated part of the egg in more detail. In this letter, Spemann suggested a kind of collaborative division of labor to Baltzer by proposing restricting the work of his Freiburg group to homospermic fertilization and leaving hybrid fertilization and the hybrid merogone approach to Baltzer’s group.^38^ In fact, one of Spemann’s students, Heinrich Schütz, made constrictions on homospermic-fertilized *Triton* eggs in 250 cases in 1922/1923 and submitted his PhD thesis in 1924. These results largely confirmed Spemann’s work from 1913/1914 (Fäßler 1997, pp. 190–191). However, it becomes clear from the archival material that Spemann himself also continued conducting constriction experiments on *Triton* at that time. A 1925 laboratory notebook shows that he was engaged in fertilization experiments and constrictions with *Triton*.^39^ In the same years, Fankhauser, while working with Baltzer, performed similar experiments in Bern. In contrast to Spemann, Fankhauser was concerned with cytological interpretations that partially diverged from Spemann’s results (Fankhauser 1925, 1930a, b).

In 1928, Spemann published an extensive, 30-page article that summarized his and Schütz’s constriction experiments from the 1920s. Spemann now interpreted these results in light of his organizer concept. He explained the fact that in some cases the cleavage nuclei [“*Furchungskerne*”] were not able to induce normal development through the absence of the most important part after the constriction, namely, the cytoplasmic part that later becomes the center of organization, thus the organizer (Spemann 1928). He concluded that the termination of development was not caused by a restriction of the potential of the nucleus but by a deficiency in the cytoplasm. With respect to the intersection of problems of heredity and development, and in line with his epigenetic approach, he concluded: “It is not the disposition that is missing, but the impetus for its activation.”^40^

Spemann and Baltzer corresponded until the late 1930s. In one of his last letters, Baltzer reported about how he had to interrupt his visit to the International Congress of Genetics in Edinburgh during the very last days of August 1939 because of the sudden outbreak of World War II. At this time, in autumn 1939, Spemann was retired and had begun to think about writing his autobiography. In his letters to his son Ulrich, Spemann described his everyday life during these months as filled with reading classical literature and reflecting on theology and philosophy.^41^ Whereas Spemann (even after Germany attacked Poland and turned Europe into a warzone) was seemingly cautious of expressing criticism of the Nazi regime in these personal letters, Baltzer was shocked by the political developments. In October 1939, he wrote to Spemann:

It is extremely difficult for me to write today – indeed to write at all – since the ground could fall out from under my feet.… Peace in Europe seems impossible for a long time, however the fate might turn. A completely bleak future.^42^

## Conclusion: Spemann’s Fantastical Experiment, 1936

What can be concluded from Baltzer and Spemann’s collaborative research efforts? In Baltzer’s research group in Bern, an experimental system on hybrid merogones developed that substantially contributed to a newly emerging field of developmental genetics. Manfred Laubichler and Eric Davidson (2007) emphasized the historical connection between Boveri’s merogony experiments with sea urchins at the turn of the century and later nuclear transplantation experiments with frogs by Robert Briggs, Thomas King, and John Gurdon in the 1950s and early 1960s. They argued that “indirectly, the fundamental logic of the merogony experiment contributed as well to the development of nuclear transfer … and the subsequent advances in animal cloning” (Laubichler and Davidson 2007, p. 8). As I have tried to show, there was indeed a direct historical connection that lies in the work of Boveri’s student Baltzer and his group. The research on *Triton* merogones by Baltzer and Hadorn laid a conceptual basis for the nuclear transplantation experiments in the 1950s. For example, John Gurdon, who successfully cloned the first frogs through cell nuclear transplantation around 1960, saw himself as part of a larger scientific lineage that ultimately goes back to Baltzer and Boveri. Gurdon emphasized on several occasions the following scientific genealogy. Describing himself as a student of Michail Fischberg, Gurdon emphasized that Fischberg was a

student of Hadorn, himself a student of Baltzer, who studied under Spemann and Boveri …. Fischberg had done postdoctoral work in the Genetics Department in Edinburgh, headed by C.H. Waddington. At that time, a very high proportion of all who were researching in the area of developmental biology anywhere in Europe could trace some part of their training back to the lineage of Boveri in Germany. (Gurdon 2006, p. 2)

Whereas Baltzer and the group of his students turned Boveri’s “legacy” into a future-oriented research system, Spemann remained within a holistic paradigm that was more or less concerned with rejecting the mechanistic concepts of heredity that was exemplified for Spemann by Weismann and his views on the cell nucleus as determining factor. It is within this context that Spemann mentioned the so-called “fantastical” experiment in his book from 1936 (Spemann 1936, p. 135). He there described an (at the time technically impossible) experiment of transplanting completely differentiated cell nuclei into enucleated egg cells as a kind of continuation of the logic of his constriction experiments (described above), published in 1914 and 1928. In 1936, Spemann wrote:

Decisive information about this question may perhaps be afforded by an experiment which appears, at first sight, to be somewhat fantastical. It has been shown, as pointed out before, in the egg of the sea urchin (Loeb, 1894) and the newt (Spemann, 1914) that a piece of the egg protoplasm which contains no nucleus may be induced to develop, may be “fertilized,” as it were by a descendant of the fertilized egg nucleus.… Probably the same effect could be attained if one could isolate the nuclei of the morula and introduce one of them into an egg or an egg fragment without an egg nucleus. The first half of this experiment, to provide an isolated nucleus, might be attained by grinding the cells between two slides, whereas for the second, the introduction of an isolated nucleus into the protoplasm of an egg devoid of a nucleus, I see no way for the moment. If it were found, the experiment would have to be extended so that older nuclei of various cells could be used. This experiment might possibly show that even nuclei of differentiated cells can initiate normal development in the egg protoplasm. Therefore, though it seems an anticipation of exact knowledge to say that ‘every single cell possesses the whole apparatus of potencies’ (Petersen, 1922, p. 116), yet this opinion may be right. (Spemann 1936, p. 135; see also Spemann 1938, p. 211)

It would be historically misleading to view Spemann’s description of this “thought experiment” as “foreseeing” later cloning research or even as a call for the development of appropriate experimental techniques. Spemann’s emphasis at this point was not on experimental approaches but on fundamental, more philosophical perspectives. Spemann offered this thought experiment as a kind of extension of the results of his constriction experiments from the 1910s and 1920s to illustrate that, in contrast to Weismann’s view, even differentiated cell nuclei retain the potentiality for a normal development.

However, the theoretical context of these reflections included much more than simply an alternative idea about the capacity of the nucleus in the developmental process to Weismann’s view. This is made clear in the entire chapter from which this quotation comes. Spemann, who focused on the cytoplasm (as a regulatory structure) rather than the cell nucleus, discussed in this chapter the concepts of “determination” and “potency,” especially the relation between “*Systempotenz”* [potency of the system] and “*Genompotenz”* [potency of the genome] on a much more fundamental level (Spemann 1936, p. 135; Spemann 1938, p. 211). By rejecting the mechanistic (and, for Spemann, preformist) model of Weismann and by referring to holistic approaches—such as Hans Petersen’s idea of the cell as a “whole apparatus of potencies” or Walther Vogt’s description of “determination” as an active, productive process of “setting a goal” or a process to “give positive direction” (Spemann 1936, p. 136)^43^— Spemann emphasized an ontological issue central to his own holistic approach and in contrast to Weismann’s idea of parceling out and decreasing potentialities. For Spemann, development was not a matter of a primarily mechanistic or physical process that went along only with a determination and restriction of potentialities in the living being, but exactly the opposite. In contrast to the view that would describe restriction and decreased potency as the essential aspect of determination (and, hence, development), Spemann emphasized that these processes could, on the level of the system, also be described as *unfolding* potentialities. It was this discursive framing of unfolding or activation that structured how the biological problems of development and heredity were related for Spemann. For him, the relation of heredity and development had to be understood as an “epigenetic principle” (Spemann 1924a, p. 78) in which genes were activated by a superordinate whole. It is precisely here that the concept of an “organizer” fit within Spemann’s epigenetic principle and his fundamentally holistic and partly vitalistic approach.

## Footnotes

1 See, for example, Rheinberger (2000) on Alfred Kühn’s physiological developmental genetics; Rhein-berger (2010) on Carl Correns and Max Hartmann; Richmond (2007), Satzinger (2009), and Dietrich (2011) on Richard Goldschmidt’s physiological genetics; Schwerin (2004) on Hans Nachtsheim and Mendelism; Campos and Schwerin (2016) on Erwin Baur; Brandt and Schloegel (2016) on Victor Jollos and the genetics of Paramecium; Gausemeier (2005) on genetic research at the Kaiser Wilhelm Institutes during the Third Reich; and Deichmann (1992) on biologists under Hitler.

2 On a “split between embryology and genetics,” see the classical article of Allen (1986). Allen has analyzed the conversion of Thomas Hunt Morgan from an embryologist (who attacked the chromosome theories of heredity until 1910) to the most prominent representative of a Mendelian-chromosome theory, with a shift from an embryological, developmental to a genetic, transmission view of heredity in the context of growing mechanistic materialism in biology in the United States during the early twentieth century.

3 On the history of embryology, see: Haraway ([1976] 2004); Horder et al. (1986); Gilbert (1991); Maienschein (1991b, 2003); Laubichler and Maienschein (2007); Hopwood (2009, 2011).

4 Maienschein distinguishes between four main approaches in how embryologists understood organization. These are a focus on (1) “cytoplasmic localization (stressing intrinsic and structural factors),” (2) “nuclear determination (stressing predetermination and chromosomes),” (3) “metabolic and chemical organization (stressing intrinsic wholistic factors),” and (4) “organic regulation (stressing the organic whole and allowing vitalistic considerations)” (Maienschein 1997, p. 222).

5 Spemann to Roux, 15. II. 1907 („sympathischere Bezeichnung“), Universitätsbibliothek J.C. Senckenberg, Frankfurt, Spemann papers, Na 47, Nr. 4.

6 See the letters from Correns to Spemann: In February 1923, Correns wrote that Hertwig also had tried to get Correns to give a paper at the conference in Munich. From the way Correns phrased this, we can conclude that Hertwig also had asked Spemann to give that paper on heredity and *Entwicklungsmechanik*. Universitätsbibliothek J.C. Senckenberg, Frankfurt, Spemann papers, Na 47, Nr. 1.

7 When the German state was not able to pay the reparations resulting from World War I, the already existent allied occupation of the Rhineland was extended to neighboring regions. In January 1923, French and Belgian soldiers were sent to the (previously) unoccupied areas of the Ruhr River region, the industrial heart of Germany. In the months that followed, there were widespread strikes by the mine workers and others. These were financially supported by the German government in Berlin. To comply with the reparations, but also to financially support this passive resistance, the German state began to print money at an unprecedented degree, exacerbating the already ongoing inflation.

8 On Wednesday, September 26, right in the middle of the conference week, the nationalist and anti-Semitic politician Gustav Ritter von Kahr declared a kind of nationalist dictatorship in Bavaria in protest against the German *Reichsregierung* in Berlin, which had stopped supporting workers’ opposition in the Ruhr River region.

9 A detailed overview can be found in Harwood (1993, pp. 295–314); see also Ringer (1969); Hopwood (1997); Schneider (1999).

10 In addition to his career as a scientist, Spemann was also director of the *Volkshochschule* (an adult education program) in Freiburg from 1920 to 1933. Very early on, Spemann actively supported the ideas of the *Landschulheimbewegung* (a reform pedagogical movement) and was a close and life-long friend with its founder, Hermann Lietz.

11 Spemann to Huxley, 14. January 1925, Rice University, Woodson Research Center, Fondren Library, Huxley Papers, box 8, folder 4. “Lieber Herr College! Soeben habe ich Bateson geschrieben, daß ich dies Frühjahr nicht kommen kann. Zuviel Arbeit und dann – Sie würden auch keinen Vortrag in Deutschland halten, wenn wir einen Teil von England besetzt hielten und zum versprochenen Termin nicht hinausgingen. Besuchen Sie mich einmal hier. Mit besten Grüßen, Ihr ergebener H. Spemann.”

12 August Weismann had been the chair for zoology in Freiburg from 1868 to 1912. His direct successor was Franz Doflein, who moved to Breslau in 1918. Spemann received the offer in 1918 and became professor in Freiburg in April 1919 (Fäßler 1997, pp. 58–59).

13 The blastopore lip is the emerging structure in the developing embryo where the opening and infolding of tissue starts when the blastula stage turns into the gastrula stage through an invagination.

14 Fäßler (1997, pp. 227–302) gives a very detailed analysis of the experimental shifts in Spemann’s work that led to the organizer concept in the years from 1915 to 1924. See also Fäßler (1996).

15 Letter from Goldschmidt to Spemann, 29 October 1935. University Archive Freiburg, Spemann Papers, C 181, Signatur 2 (“Gratulationen in Folge des Nobelpreis”).

16 This quotation is from the American zoologist Frank Lillie (Letter from Lillie to M.H. Jacobs, 18. May 1931, quoted in Fäßler 1997, p. 1).

17 In the first sentences, Spemann began by critically noting that the term “*Vererbung*” [inheritance] would introduce semantic ambiguity into biology because of the ambiguities in its everyday usage. The biological notion could only make sense with respect to the inheritance of *single* characteristics from ancestor to descendants (like the inheritance of “goods and chattels” [*Hab und Gut*]). The term would lose any explanatory power when it comes to accounting for the emergence of the whole organism (Spemann 1924a, p. 65). On the history of heredity and the metaphorical origins of the term in the nineteenth century, see Müller-Wille and Rheinberger (2012); on the history of genetics and hereditary studies, see Müller-Wille and Richmond (2016); for a general overview of the variety of approaches in hereditary studies and genetics from 1850 to 1930, see the contributions in Müller-Wille and Brandt (2016).

18 See also Brandt, “Vitalism, Holism, and Metaphorical Dynamics of Hans Spemann’s ‘Organizer’ in the Interwar Period,” in this issue.

19 “eine faktorentopographische Chromosomenkarte Morgans mutet an wie ein mit konkretem Inhalt ausgefülltes Weismannsches Schema” (Spemann 1924a, p. 67).

20 On Goldschmidt, see, for example, Richmond (2007); Satzinger (2009); Dietrich (2011).

21 “Auch hierin entwickelten sich unsere Vorstellungen im Anschluß an Weismannsche Ideen, wenn auch in diesem Fall von Anfang an im Gegensatz zu ihnen. Nach Weismann sind die Determinanten … im Keimplasma, d.h. in den Chromosomen des befruchteten Eies zu einem äußerst komplizierten Bau vereinigt, der im Ablauf der erbungleichen Kernteilungen in strenger Gesetzmäßigkeit in seine Bestandteile zerfällt und in genau festgelegter Reihenfolge die einzelnen Determinanten freigibt. […]. Danach leitet also das Chromatin allein die ganze Entwicklung vom Anfang bis zum Ende, ihr Ablauf ist dem eines äußerst feinen, aber starren Uhrwerks zu vergleichen. […] Jene rein evolutionistischen Vorstellungen Weismanns sind heute wohl allgemein aufgegeben. Am entscheidensten wurden sie durch Experimente Boveris getroffen, durch welche ihre Grundlage, die Möglichkeit erbungleicher Kernteilung, entzogen wurde“ (Spemann 1924a, pp. 73–74).

22 This will be discussed in more detail below.

23 “Hier liegt bei mancher Verwandtschaft der Unterschied gegen unsere heutigen Vorstellungen, der im letzten Grunde damit zusammenhängt, daß Weismann extremer Evolutionist war, während wir zu mehr epigenetischen Vorstellungen zurückgekehrt sind“ (Spemann 1924a, p. 67).

24 “In den Vorlesungen habe ich einen Vergleich gebraucht, der das Verhalten von Plasma und Kern deutlich machen sollte, der aber, wie alle Vergleiche – vielleicht mehr als gewöhnliche Vergleiche – hinkt, nämlich des Plasmas mit einem Maschinengewehr und der Gene mit dessen Patronen. Das tertium comparationis ist, daß Gene und Patronen von außen zur Wirkung gebracht werden, daß sie in bestimmter Reihenfolge und Zeitfolge daran kommen, und die Notwendigkeit, daß Gene und Patronen in den Mechanismus passen. Der Vergleich hinkt aber, wie schon gesagt, in mehrfacher Hinsicht, vor allem darin, daß die Reihenfolge der Patronen durch das Band, auf dem sie befestigt sind, und nicht durch das Gewehr bestimmt ist, und ebensosehr darin, daß bei ihnen keine quantitative Beeinflussung der Wirkung von außen möglich ist, und es sich bei dem Einpassen der Patronen nur um ein Entweder-oder handelt" (Correns 1937, pp. 127–128).

25 Letter of Correns to Spemann, 11 February 1923, Universitätsbibliothek J.C. Senckenberg, Frankfurt, Spemann Papers, Na47, Nr.1: “Was Sie über die ‘Organisatoren’ schreiben, hat mich SEHR interessiert. Wenn ich Sie recht verstanden habe, empfängt das große taeniatus-Stück den (fortdauernden?) Anstoß zur Entwicklung von dem kleinen cristatus-Stück, bleibt aber artspezisisch, wie dieses. Es ist der Gegensatz zwischen dem, was man als Gene in den Chromosomen lokalisiert annimmt, und dem, was für die Entfaltung der Gene in der richtigen Reihenfolge sorgt, den Patronen und dem Maschinengewehr. Habe ich Sie wohl recht verstanden?”

26 On the skepticism regarding the approach of *Drosophila* genetics among German biologists (especially zoologists), see Harwood (1993, pp. 49–98).

27 For the biographical and institutional details of this period, see Fäßler (1997, pp. 21–58).

28 The second assistant in Spemann’s Freiburg group was Otto Mangold, who completed his PhD in 1919 under Spemann’s supervision. Mangold contributed mainly to the research on the organizer, and he later succeeded Spemann at the Kaiser Wilhelm Institute in Berlin and, from 1937 to 1945, also as the chair in Freiburg. After the war, Mangold was suspended because of his political activities during the Nazi period.

29 Spemann, Gedächtnisrede auf Theodor Boveri, gehalten am 3. Februar 1916 (Spemann 1916). University Archive Freiburg, Spemann Papers, C 181, Signatur 104.

30 The book is based on the Silliman Lectures, which Spemann gave at Yale University in October 1933. The book was published in Germany in 1936; the English translation with a slightly different title (*Embryonic Development and Induction*), published two years later in 1938, was also dedicated to Theodor Boveri.

31 Spemann and Baltzer exchanged letters from around about 1918 until the late 1930s, and their correspondence is among the very few that uses the German “Du” (after 1922), Universitätsbibliothek J.C. Senckenberg, Frankfurt, Spemann Papers, Na47, Nr. 1.

32 Unpublished manuscript: Hans Spemann, Der Entdecker des Organisators im tierischen Ei. 12. September 1941. Von F. Baltzer, Bern. University Archive Freiburg, Spemann Papers, C 181, Signatur 129: “das bernische Zoologische Institut steht bei Spemann in tiefer Dankesschuld. Wir sind – sei es direkt oder indirekt in ‘zweiter Generation’ – fast alle seine Schüler gewesen.”

33 Satzinger (2009, p. 114) refers to a letter from Boveri to Spemann, 27 November 1913.

34 “Wenn wir heute ein Werk über Vererbungslehre durchblättern, sehen wir das großartige Bild vor uns entrollt, zu dem die Zucht der reinen Linien und die Mendelforschung die Grundlinien geliefert haben. Wir lernen die Lehre von den Genen oder Erbeinheiten kennen, die in immer neuen Kombinationen die Kette der Individuen durchziehen.… Über die Vererbung selbst aber, d.h. über die Frage, wie die in der Zygote gegebene Konstellation zu jenem Erbeffekt hinführt, mit dem der Vererbungsforscher arbeitet, darüber erfahren wir nichts; denn darüber wissen wir eben trotz aller exakten Vererbungslehre so gut wie nichts” (Boveri [1918, p. 365]; see also Satzinger [2009, p. 116]).

35 “Es liegt also am Eiplasma, nicht an den Kernen, zu welchem Teile des Embryos sich die Teile des Keimes entwickeln” (Spemann 1914, p. 219).

36 For a detailed historical analysis of these debates, see Harwood (1993) and Sapp (1987). East (1934) provides a contemporary overview on the nucleus–cytoplasm debate from the perspective of a US scientist who favored *Kernmonopol* [the monopoly of the nucleus].

37 Letter from Baltzer to Spemann 29 October 1922, Universitätsbibliothek J.C. Senckenberg, Frankfurt, Spemann Papers, Na 47, Nr. 1: “Vom Merogon muss ich noch erzählen: Er erwies uns die Freundlichkeit, zu metamorphosieren & präsentierte sich am hiesigen Schweiz. Naturforschertag ganz munter. Dann aber protestierte er und ertrank. Er war wohl zu dumm, nach Entschrumpfung der Kiemen aus dem Wasser zu gehen & wir waren zu dumm, ihm dasselbe zeitig genug wegzunehmen. Man kann die Frage der Entwicklungsfähigkeit der homospermen Haploide wohl so beantworten: Die Lebensfähigkeit ist sicher geringer und es wird von Interesse sein, ob man durch histog. + topographische Untersuchung der Schnitte etwas über die Ursachen finden kann. Trotzdem glaube ich, dass man einige Exemplare weiter kriegt. Wir haben nun die Zuchttechnik besser gelernt, haben von normalen viele Schwächlinge durchgebracht.”

38 Letter of Spemann to Baltzer, 13 April 1922, Universitätsbibliothek J.C. Senckenberg, Frankfurt; quoted from Fäßler (1997, p. 190).

39 Laboratory Notebook “Triton 1925,” University Archive Freiburg, Spemann Papers, C 181, Signatur 57.

40 “Es fehlt also nicht die Anlage dazu, sondern der Anstoß zu ihrer Betägigung” (Spemann 1928, p. 132).

41 Correspondence Hans and Ulrich Spemann 1938-1940, University Archive Freiburg, Spemann Papers, C 0181, Signatur 7.

42 Letter from Baltzer to Spemann, 8. October 1939, Universitätsbibliothek J.C. Senckenberg, Frankfurt, Spemann Papers, Na 47, Nr. 1: “Es ist mir heute so ungeheuer schwer zu schreiben, – überhaupt zu schreiben – weil mir der Boden unter den Füßen abtauchen könnt. Die Auseinandersetzung zwischen den beiden Machtgruppen Europas vollzieht sich in einer.… Weise, dass mir eine ‘Befriedung’ Europas auf lange Zeit unmöglich erscheint, das Schicksal mag sich wenden, wie es will. – Eine vollkommen trübe Zukunft.”

43 Walther Vogt (1888–1941) was an anatomist from the Universities of Munich and Zurich who belonged to those biologists outside of the Spemann school whose research developed along similar lines to the work in Freiburg; Hans Petersen (1885–1946) was a former student of the anatomist Hermann Braus (1868–1924), a friend of Spemann’s from his days in Würzburg and Braus’s successor as professor of anatomy at the University of Würzburg. Spemann referred to extensive quotations from both in this chapter.
